# B Lymphocytes, but Not Dendritic Cells, Efficiently HIV-1 *Trans* Infect Naive CD4^+^ T Cells: Implications for the Viral Reservoir

**DOI:** 10.1128/mBio.02998-20

**Published:** 2021-03-09

**Authors:** Abigail Gerberick, Diana C. DeLucia, Paolo Piazza, Mounia Alaoui-El-Azher, Charles R. Rinaldo, Nicolas Sluis-Cremer, Giovanna Rappocciolo

**Affiliations:** a Department of Infectious Diseases and Microbiology, Graduate School of Public Health, University of Pittsburgh, Pittsburgh, Pennsylvania, USA; b Division of Infectious Diseases, Department of Medicine, University of Pittsburgh School of Medicine, Pittsburgh, Pennsylvania, USA; c Department of Pathology, University of Pittsburgh School of Medicine, Pittsburgh, Pennsylvania, USA; University of Michigan Medical School; Rutgers-Robert Wood Johnson Medical School

**Keywords:** B lymphocytes, HIV-1, dendritic cells, naive CD4^+^ T cells, *trans* infection

## Abstract

Insight into the establishment and maintenance of HIV-1 infection in resting CD4^+^ T cell subsets is critical for the development of therapeutics targeting the HIV-1 reservoir. Although the frequency of HIV-1 infection, as quantified by the frequency of HIV-1 DNA, is lower in CD4^+^ naive T cells (T_N_) than in the memory T cell subsets, recent studies have shown that T_N_ harbor a large pool of replication-competent virus. Interestingly, however, T_N_ are highly resistant to direct (*cis*) HIV-1 infection *in vitro*, in particular to R5-tropic HIV-1, as T_N_ do not express CCR5. In this study, we investigated whether T_N_ could be efficiently HIV-1 *trans* infected by professional antigen-presenting B lymphocytes and myeloid dendritic cells (DC) in the absence of global T cell activation. We found that B cells, but not DC, have a unique ability to efficiently *trans* infect T_N_ *in vitro*. In contrast, both B cells and DC mediated HIV-1 *trans* infection of memory and activated CD4^+^ T cells. Moreover, we found that T_N_ isolated from HIV-1-infected nonprogressors (NP) harbor significantly disproportionately lower levels of HIV-1 DNA than T_N_ isolated from progressors. This is consistent with our previous finding that antigen-presenting cells (APC) derived from NP do not efficiently *trans* infect CD4^+^ T cells due to alterations in APC cholesterol metabolism and cell membrane lipid raft organization. These findings support that B cell-mediated *trans* infection of T_N_ with HIV-1 has a more profound role than previously considered in establishing the viral reservoir and control of HIV-1 disease progression.

## Abstract (precis)

The latent human immunodeficiency virus type 1 (HIV-1) reservoir in persons on antiretroviral therapy (ART) represents a major barrier to a cure. Although most studies have focused on the HIV-1 reservoir in the memory T cell subset, replication-competent HIV-1 has been isolated from T_N_, and CCR5-tropic HIV-1 has been recovered from CCR5^neg^ T_N_ from ART-suppressed HIV-1-infected individuals.

## INTRODUCTION

Latently infected resting CD4^+^ T cells constitute a major reservoir of persistent HIV-1 infection. Strategies that lead to a significant reduction or elimination of this reservoir could help in the development of either a functional or sterilizing cure (1–4). The CD4^+^ T cell population is heterogeneous, broadly comprised of naive (T_N_) and memory cells that differ in life span, proliferative capacity, localization, and HIV-1 coreceptor expression. Memory cells are further categorized by various stages of differentiation, namely, central memory (T_CM_), transitional memory, and effector memory. The latent HIV-1 reservoir in memory T cell subsets has been extensively studied, whereas T_N_ have been largely overlooked (5, 6). Although resting T_N_ are highly resistant to direct, *cis* infection with HIV-1 *in vitro*, we and others have shown that HIV-1 DNA is detectable in T_N_ of viremic and virus-suppressed individuals (7, 8). While the frequency of HIV-1 infection in T_N_ is lower than that in T_CM_, as much or more virus is produced by T_N_ after reactivation with latency-reversing agents (LRAs) (9). Moreover, paradoxically, CCR5-tropic HIV-1 has been recovered from T_N_ despite the fact that they do not express the CCR5 coreceptor (9–12).

HIV-1 can infect target cells via direct, *cis* infection or through a cell-to-cell transfer which can result in *trans* infection (13–15). The latter mechanism has been extensively described as mediated by professional antigen-presenting cells (APC), i.e., monocytes/macrophages, myeloid dendritic cells (DC), and B lymphocytes. Indeed, HIV-1 *trans* infection mediated by APC is 10- to 1,000-fold more efficient than passive, *cis* dissemination of virions through the extracellular milieu (16, 17). We have previously shown that APC derived from HIV-1-infected nonprogressors (NP) do not efficiently transfer HIV-1 to CD4^+^ T cells due to alterations in APC cholesterol metabolism and cell membrane lipid raft organization (18, 19). In the present study, we show that B lymphocytes, but not DC, have the exclusive ability to efficiently *trans* infect T_N_ with CCR5-tropic HIV-1. Furthermore, T_N_ isolated from NP harbor significantly lower levels of HIV-1 DNA than do T_N_ isolated from HIV-1 progressors (PR). These findings support that B cell-mediated *trans* infection of T_N_ with HIV-1 has a more profound role than previously considered in establishing the viral reservoir and control of HIV-1 disease progression.

## RESULTS

### B cells *trans* infect CD4^+^ T cells with high efficiency.

**FIG 1:**
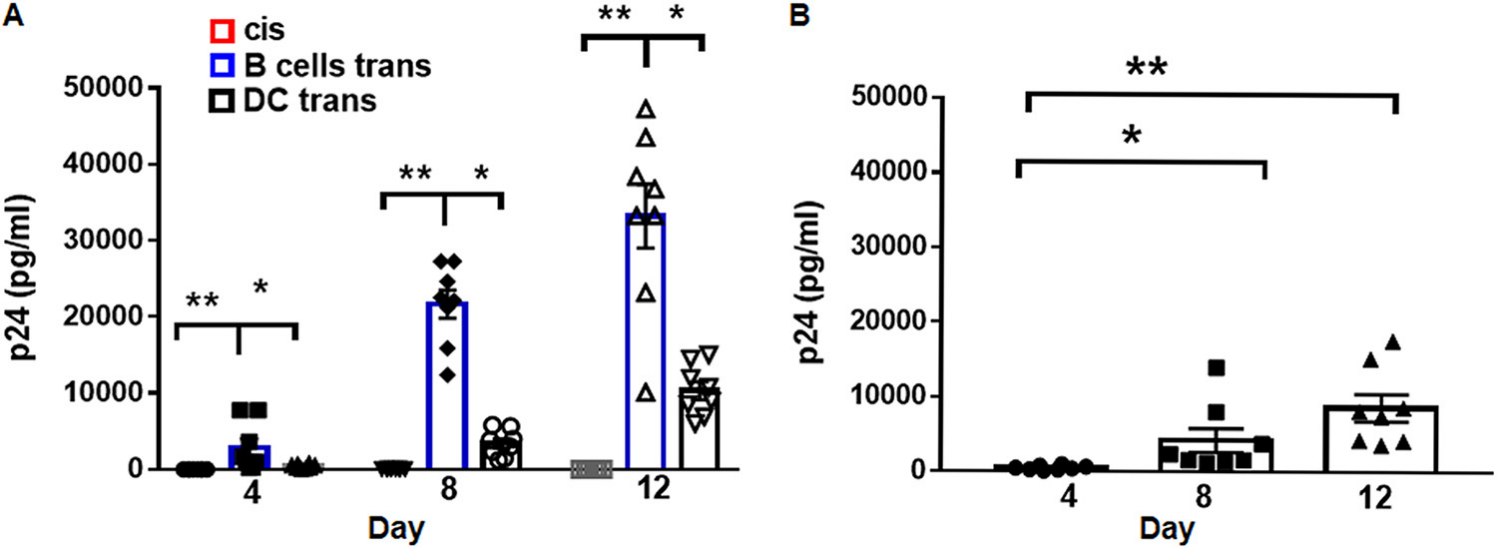
B cells *trans* infect CD4^+^ T cells with higher efficiency than DC. (A) B cells or immature DC (iDC) were pulsed with HIV-1^BaL^ (10^−3^ MOI) and cocultured with PHA/IL-2-activated autologous CD4^+^ T cells at a 1:10 ratio for up to 12 days as described in Materials and Methods (*trans* infection). PHA/IL-2-activated CD4^+^ T cells were also pulsed with HIV-1^BaL^ (10^−3^ MOI) and cultured alone (*cis* infection). Coculture supernatants were tested at the indicated time points for HIV-1 Gag p24 levels by ELISA. (B) PHA/IL-2-activated CD4^+^ T cells were pulsed with HIV-1^BaL^ (10^−1^ MOI) and cultured up to 12 days. Culture supernatants were tested at the indicated time points for HIV-1 Gag p24 levels by ELISA. Data are mean ± SE; *n* = 8; *, *P* < 0.05; **, *P* < 0.005.

B cells activated with CD40L and interleukin-4 (IL-4), which mimics signals received from activated CD4^+^ T cells, express the C-type lectin DC-specific intercellular adhesion molecule-3-grabbing nonintegrin (DC-SIGN) and can capture HIV-1, leading to *trans* infection of CD4^+^ T cells (15, 20). In this study, we first extended this finding by demonstrating that B cells or DC loaded with a low, 10^−3^ multiplicity of infection (MOI) of R5-tropic HIV-1^BaL^ could *trans* infect phytohemagglutinin (PHA)/IL-2-activated CD4^+^ T cells, with the efficiency of *trans* infection being significantly greater for B cells than DC (one-way analysis of variance [ANOVA], *P* < 0.0001, Fig. 1A). This is of importance because, as we showed previously, only about 10 to 15% of activated B cells express DC-SIGN, compared to 100% of DC, therefore potentially implicating B cells as more efficient than DC in mediating HIV-1 *trans* infection. In agreement with our previous findings (15), CD4^+^ T cells were refractory to *cis* HIV-1 infection at the same low, 10^−3^ MOI but were productively infected with a 100-fold-greater dose of 10^−1^ MOI (Fig. 1B). We conclude from these data that B lymphocytes, activated by two surrogates for CD4^+^ T helper cells, i.e., IL-4 and CD40L, are more efficient than myeloid DC in mediating HIV-1 *trans* infection of activated CD4^+^ T lymphocytes.

### B cells, but not DC, *trans* infect CD4^+^ naive T cells *in vitro*.

**FIG 2:**
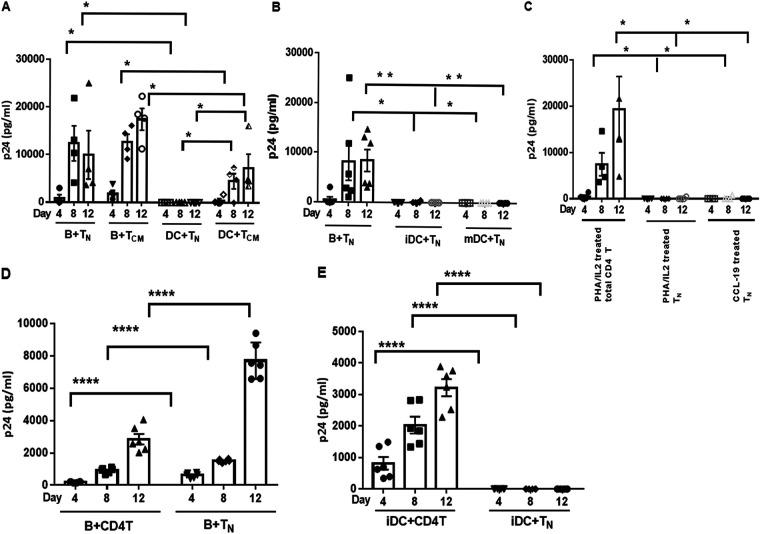
Only B cells *trans* infect T_N_. (A) B cells or iDC were pulsed with HIV-1^BaL^ (10^−3^ MOI) and cocultured with PHA/IL-2-activated purified naive (T_N_; *n* = 5) or central memory (T_CM_; *n* = 4) CD4^+^ T cells. Coculture supernatants were tested at the indicated time points for HIV-1 Gag p24 levels by ELISA. Data are mean ± SE. (B) T_N_ were treated with CCL-19, washed, and cocultured with HIV-1^BaL^ (10^−3^ MOI)-pulsed B cells, iDC, or mature DC (mDC). Coculture supernatants were tested at the indicated time points for HIV-1 Gag p24 by ELISA. Data are mean ± SE, *n* = 6. (C) Total CD4^+^ T cells or T_N_ were treated with PHA/IL-2 or CCL-19, pulsed with HIV-1^BaL^ (10^−1^ MOI), and cultured alone. Culture supernatants were tested at the indicated time points for HIV-1 Gag p24 by ELISA. Data are mean ± SE, *n* = 4. (D) B cells pulsed with HIV-1 92FR_BX08 (10^−3^ MOI) were cocultured with CCL-19-treated total CD4^+^ T cells or T_N_ for up to 12 days. Coculture supernatants were tested at the indicated time points for HIV-1 Gag p24 by ELISA. Data are mean ± SE, *n* = 3 replicates from 2 donors. (E) iDC pulsed with HIV-1 92FR_BX08 (10^−3^ MOI) were cocultured with CCL-19-treated total CD4^+^ T cells or T_N_ for up to 12 days. Coculture supernatants were tested at the indicated time points for HIV-1 Gag p24 by ELISA. Data are mean ± SE, *n* = 3 replicates from 2 donors. Data were analyzed by one-way ANOVA followed by *ad hoc* Student’s *t* test. *, *P* < 0.05; **, *P* < 0.005; ***, *P* < 0.0005; ****, *P* < 0.0001.

T_N_ do not express CCR5; however, *in vivo*, T_N_ harbor R5-tropic HIV-1 (10, 11, 21, 22). We therefore hypothesized that T_N_ are infected through an APC-mediated *trans* infection mechanism that does not require CCR5 expression by the T cells. To test this hypothesis, we used purified T_N_ and T_CM_ as targets for *trans* infection mediated by autologous B lymphocytes or DC that were loaded with 10^−3^ MOI of R5-tropic HIV-1^BaL^. Consistent with the approach described in Fig. 1, we initially used PHA/IL-2-activated CD4^+^ T_N_ and T_CM_ as targets. B cells were able to productively *trans* infect both T_N_ and T_CM_ with R5-tropic HIV-1, whereas DC were able only to *trans* infect the T_CM_ subset (Fig. 2A). As previously shown by *trans* infection of total CD4^+^ T cells, B cell-mediated *trans* infection of both T_N_ and T_CM_ was significantly more productive than DC-mediated *trans* infection of either subset (one-way ANOVA, *P* < 0.0001).

PHA/IL-2 treatment of T_N_ and T_CM_ induces T cell activation, thus rendering them more susceptible to HIV-1 infection. Therefore, we next assessed B cell- and DC-mediated HIV-1^BaL^ *trans* infection of T_N_ treated with the chemokine CCL-19. As described previously (12), CCL-19 neither elicits T cell activation nor induces CCR5 or CXCR4 expression, but enhances *cis* HIV-1 infection of resting CD4^+^ T cells. We found that only B cells were able to *trans* infect CCL-19-treated T_N_, resulting in detectable HIV-1 Gag p24 in the coculture supernatants (Fig. 2B). In contrast, neither mature DC (mDC) nor immature DC (iDC) mediated *trans* infection, indicating that the ability of DC to *trans* infect T_N_ did not depend on their maturation status (one-way ANOVA, *P* < 0.0001). As expected, T_N_ were refractory to direct *cis* infection of HIV-1^BaL^ using either PHA/IL-2- or CCL-19-conditioned medium, while total CD4^+^ T cells were susceptible to productive *cis* infection (Fig. 2C). Our findings were further confirmed using an R5-tropic patient isolate, HIV-1 BX08(92FR_BX08), obtained from the NIAID AIDS Reagent Repository (23). We showed that B cells could efficiently *trans* infect both total CD4^+^ T cells and T_N_, while iDC could *trans* infect only total CD4^+^ T cells (Fig. 2D and E). *Cis* infection of T_N_ was undetectable (not shown). Taken together, these results support that B lymphocytes have a unique ability to mediate highly productive *trans* infection of naive CD4^+^ T cells with R5-tropic HIV-1.

### Coculture with B cells or DC does not affect the T_N_ phenotype.

**FIG 3:**
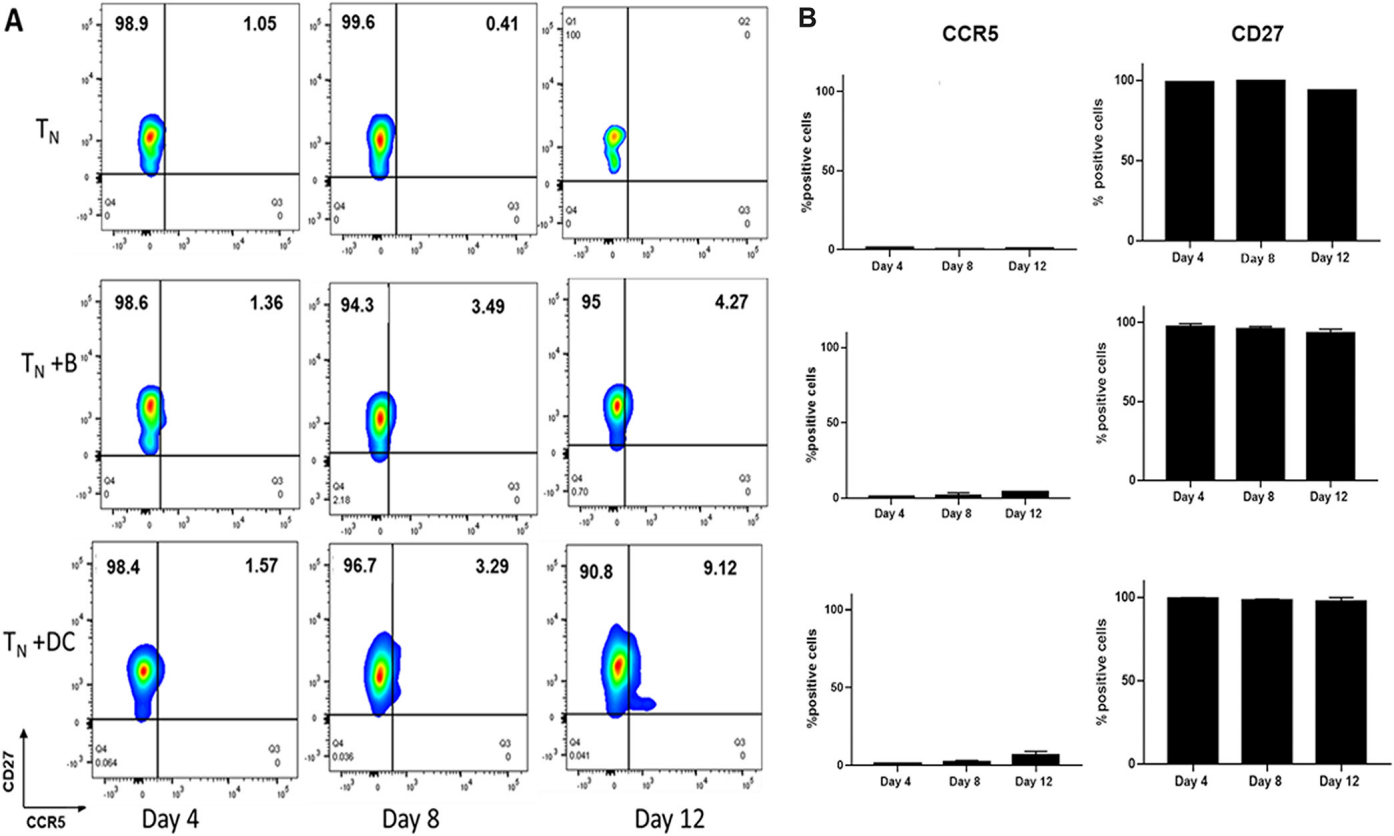
Coculture with B cells or DC does not affect T_N_ phenotype. (A) CCL-19-treated T_N_ were cultured alone (top row) or cocultured with HIV-1^BaL^ (10^−3^ MOI)-pulsed B cells or iDC (middle and bottom rows, respectively), sampled at the indicated time points, stained with anti-CCR5 and CD27 MAb, and analyzed by fluorescence-activated cell sorting (FACS) as described in Materials and Methods. Representative data from 3 independent experiments. (B) CCR5 and CD27 percent positive cells in control T_N_ cultures or cocultures. Data are mean± SE, *n* = 3.

To address whether the CD4^+^ T_N_ phenotype was altered through coculture with the APC, potentially affecting their efficiency of being infected with HIV-1, we analyzed T_N_ for CCR5 and CD27 expression. T_N_ cultured alone served as a control. CD27 expression was chosen instead of CCR7 expression because CCL-19 can induce downregulation of CCR7 (24). The flow cytometry gating strategy is shown in Fig. S1 of the supplemental material. Neither B cells nor DC induced a significantly higher expression of CCR5 up to 12 days in culture, although we detected a slight increase of CCR5 between day 8 and 12 in the DC-T_N_ cocultures (Fig. 3A). Expression of the CD27 marker also remained unchanged throughout the coculture period (Fig. 3B).

10.1128/mBio.02998-20.1 FIG S1 Gating strategy to determine T_N_ phenotype. Lymphocytes were gated first based on forward and side scatter, followed by doublet event exclusion, then by exclusion of dead cells (Aqua dye positive). CD4^+^ positive cells were then gated into CD45RA-negative and CD45RA-positive populations, with the latter population being 100% CCR7 positive as well as CCR5 negative. Download FIG S1, TIF file, 1.1 MB. Copyright © 2021 Gerberick et al. 2021 Gerberick et al. https://creativecommons.org/licenses/by/4.0/ This content is distributed under the terms of the Creative Commons Attribution 4.0 International license.

These data show that there is no significant alteration of the T_N_ phenotype during coculture with APC and confirm that B cells can establish HIV-1^BaL^ infection in T_N_ in the absence of significant CCR5 coreceptor expression.

### Detection of intracellular HIV-1 p24 in APC-T_N_ cocultures.

**FIG 4:**
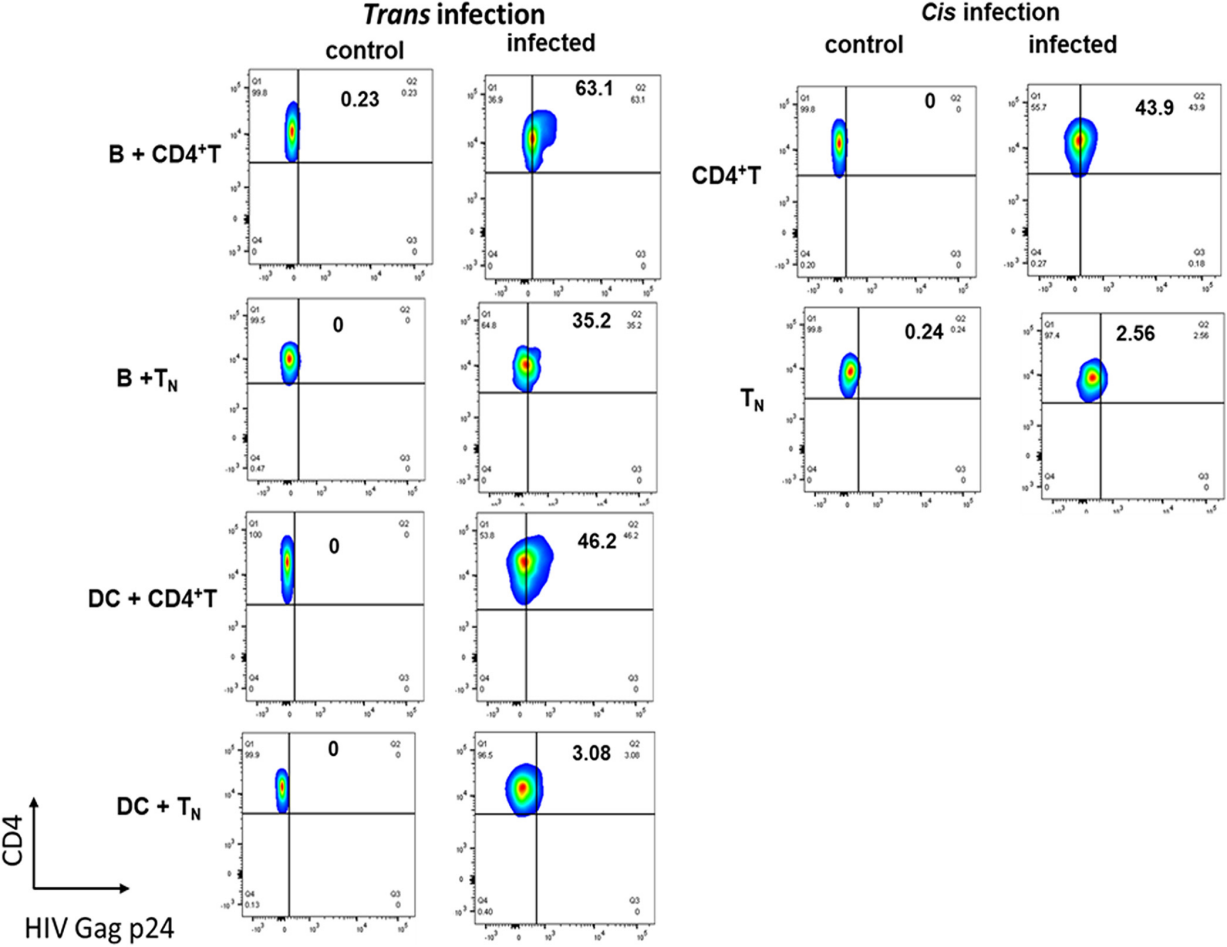
Detection of HIV-1 p24 antigen in *trans* infection coculture with B cells or DC. (Left) *Trans* infection. B cells or iDC were pulsed with HIV-1^BaL^ (10^−3^ MOI) and cocultured with CCL-19-treated total CD4^+^ T cells or T_N_. Cocultures were sampled after 8 days, stained with anti-Kc57, -CD4, -CD3, or -CD19, and analyzed by FACS as described in Materials and Methods. (Right) *Cis* infection. CCL-19-treated total CD4^+^ T cells or T_N_ were pulsed with HIV-1^BaL^ (10^−1^ MOI) and cultured alone. Cultures were sampled after 8 days, stained, and analyzed by flow cytometry in parallel to the *trans* infection cocultures. Controls represent uninfected cultures. Representative data from 2 independent experiments.

We next questioned if the detection of HIV-1 Gag p24 that we measured in the *trans* infection coculture supernatants reflected p24 intracellular localization. We therefore stained cells collected from the *trans* infection wells and examined them for HIV-1 p24 expression by flow cytometry. We were able to detect intracellular HIV-1 p24 in the cocultures of B cells with either total CD4^+^ T cells or T_N_ (Fig. 4, left). In the DC-mediated *trans* infection cocultures, we could detect HIV-1 p24 only in the DC-total CD4^+^ T cell wells, with very low levels in the DC-T_N_ cocultures. As shown in Fig. 4 (right), only total CD4^+^ T cells supported direct, *cis* infection with HIV-1. Taken together, these data further support the conclusion that only B lymphocytes can efficiently *trans* infect T_N_.

### B cell *trans* infection of T_N_ is mediated by DC-SIGN.

**FIG 5:**
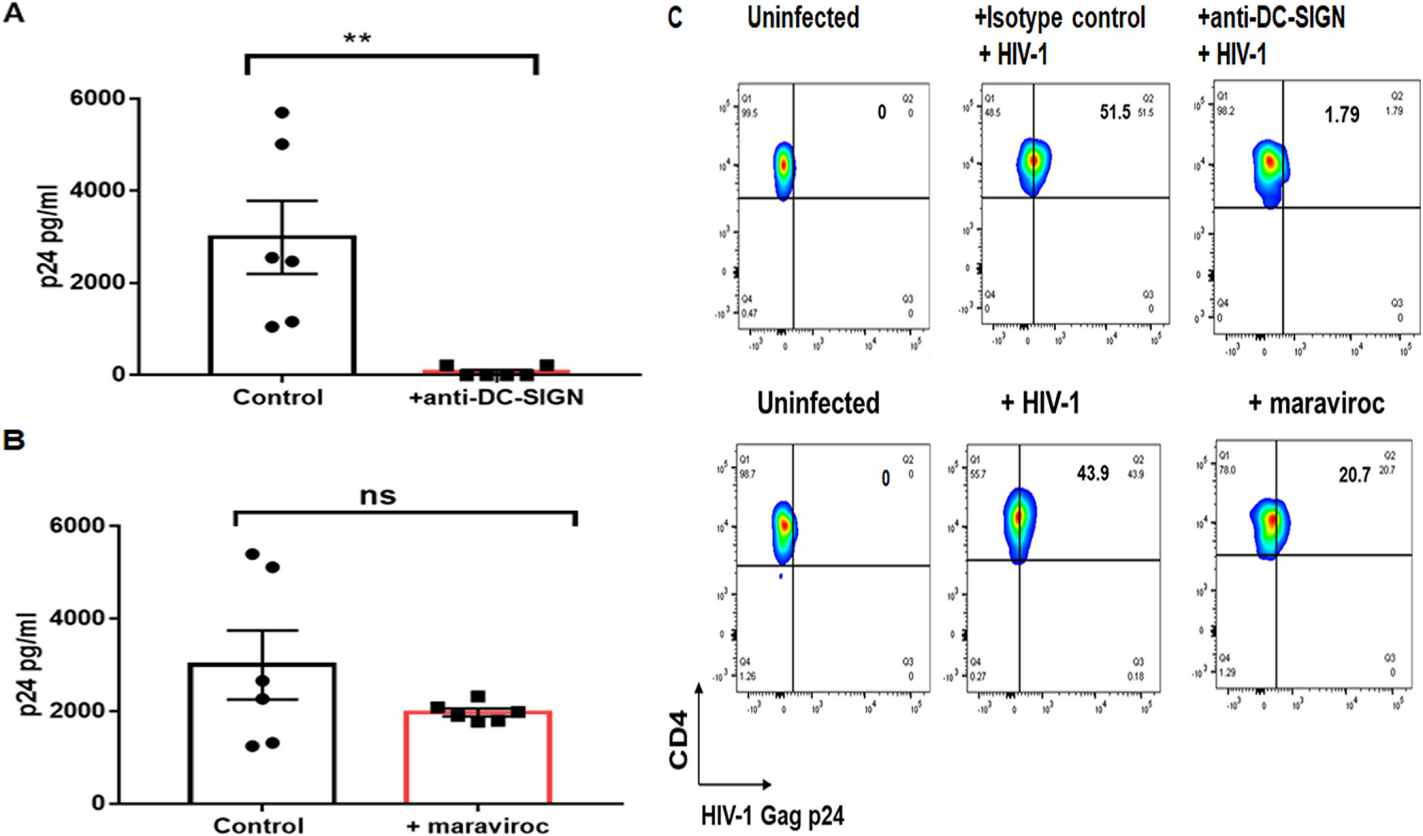
B cell-mediated *trans* infection of T_N_ is inhibited by anti-DC-SIGN. (A) B cells were incubated with 20 μg/ml anti-DC-SIGN MAb for 30 min at 4°C prior to pulsing with HIV-1^BaL^ (10^−3^ MOI) and then cocultured with CCL-19-treated T_N_ for *trans* infection as described in Materials and Methods. Supernatants were collected after 12 days and tested for HIV-1 Gag p24 by ELISA. B cells treated with mouse IgG (20 μg/ml) were used as the control. Data are mean ± SE, *n* = 3 replicates from 2 donors. (B) In parallel cultures, CCL-19-treated T_N_ were subsequently treated with maraviroc (1 μM) as described in Materials and Methods and cocultured with HIV-1^BaL^ (10^−3^ MOI)-pulsed B cells for *trans* infection. Supernatants were collected after 12 days and tested for HIV-1 Gag p24 by ELISA. Untreated T_N_ were used in the control cocultures. Data are mean ± SE, *n* = 3 replicates from 2 donors. Data were analyzed by one-way ANOVA followed by *ad hoc* Student’s *t* test. **, *P* < 0.005; ns, not significant. (C) Anti-DC-SIGN- or maraviroc-treated cells and the corresponding controls from the cocultures in panels A and B, as well as uninfected cocultures, were sampled after 12 days, stained with anti-Kc57, -CD4, -CD3, or -CD19, and analyzed by FACS as described in Materials and Methods.

We have previously shown that B cell-mediated *trans* infection of CD4^+^ T cells is inhibited by blocking of DC-SIGN (15). Therefore, we tested if DC-SIGN was also necessary to mediate *trans* infection of T_N_. Treatment of B cells with anti-DC-SIGN monoclonal antibody (MAb) significantly inhibited *trans* infection of T_N_ (Fig. 5A). We also treated T_N_ with maraviroc, a CCR5 antagonist, to determine if CCR5 expressed by T_N_ in the *trans* infection cocultures could be responsible for the infection (Fig. 5B). The concentration of maraviroc used was previously determined to significantly inhibit direct, *cis* infection of total CD4^+^ T cells, where expression of CCR5 is significantly higher than the very low or negative expression of CCR5 in T_N_ alone (25). Production of HIV-1 Gag p24 detected in the maraviroc-treated coculture supernatants was not significantly different from the untreated cocultures at the same time point. HIV-1 infection is known to induce downregulation of CD4 surface expression. Therefore, we analyzed the effect of maraviroc treatment on the CD3^+^ CD4^neg^ cell population in the cocultures by flow cytometry (Fig. S3). As shown in Fig. S3, the levels of HIV-1 p24 detected in this subpopulation were not significantly affected by maraviroc treatment. Blocking of HIV-1 *trans* infection by anti-DC-SIGN and minimal inhibition by maraviroc were confirmed by HIV-1 p24 intracellular staining of the *trans* infection cocultures (Fig. 5C).

10.1128/mBio.02998-20.3 FIG S3 Expression of HIV-1 p24 in CD3^+^ CD4^neg^ maraviroc-treated cells. Cells from maraviroc-treated cocultures or controls analyzed in Fig. 5C were further analyzed by gating on the CD3^+^ CD4^neg^ subpopulation and evaluated for HIV-1 p24 expression as described in Materials and Methods. Download FIG S3, TIF file, 0.5 MB. Copyright © 2021 Gerberick et al. 2021 Gerberick et al. https://creativecommons.org/licenses/by/4.0/ This content is distributed under the terms of the Creative Commons Attribution 4.0 International license.

### Reactivation of HIV-1 from T_N_.

**FIG 6:**
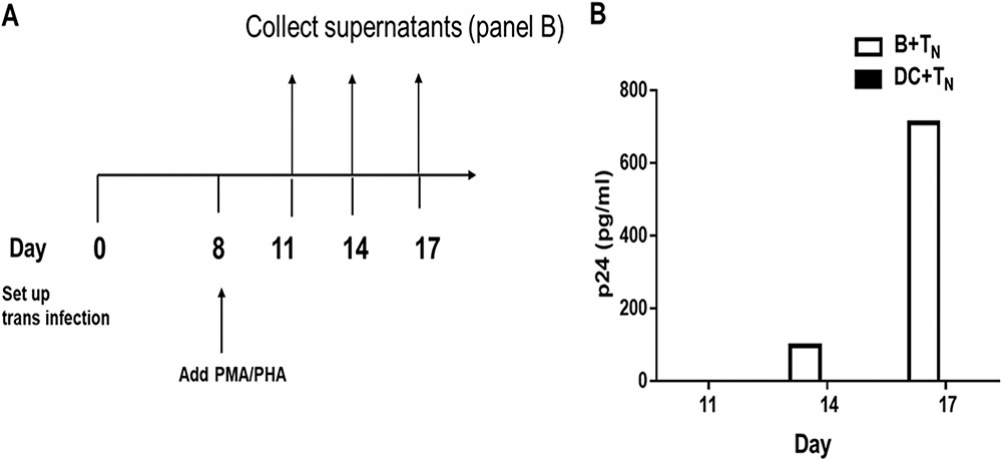
Detection of virus after LRA reactivation. (A) Schematic representation of the experimental approach to measure reversal of HIV-1 latency in T_N_ *trans* infected by B cells or iDC. (B) Cocultures were treated with LRAs at day 8 and then sampled at the indicated time points after reactivation. Supernatants were tested for HIV-1 Gag p24 by ELISA.

To assess whether HIV-1 *trans* infection of T_N_ mediated by B cells or DC resulted in HIV-1 latency, T_N_ were cultured with either 10^−3^ MOI HIV-1^BaL^-loaded B cells or DC for 8 days and then treated with phorbol myristate acetate (PMA)/PHA. Coculture supernatants were then harvested every 3 days for HIV-1 Gag p24 analysis (Fig. 6A). HIV-1 was recovered only from the B-T_N_ cocultures (Fig. 6B). This indicates that the lack of detectable virus replication in the DC-T_N_ cocultures was not due to the establishment of latency without detectable viral replication in the T_N_.

### CD4^+^ T_N_ from HIV-1-infected NP harbor less total HIV-1 DNA.

**FIG 7:**
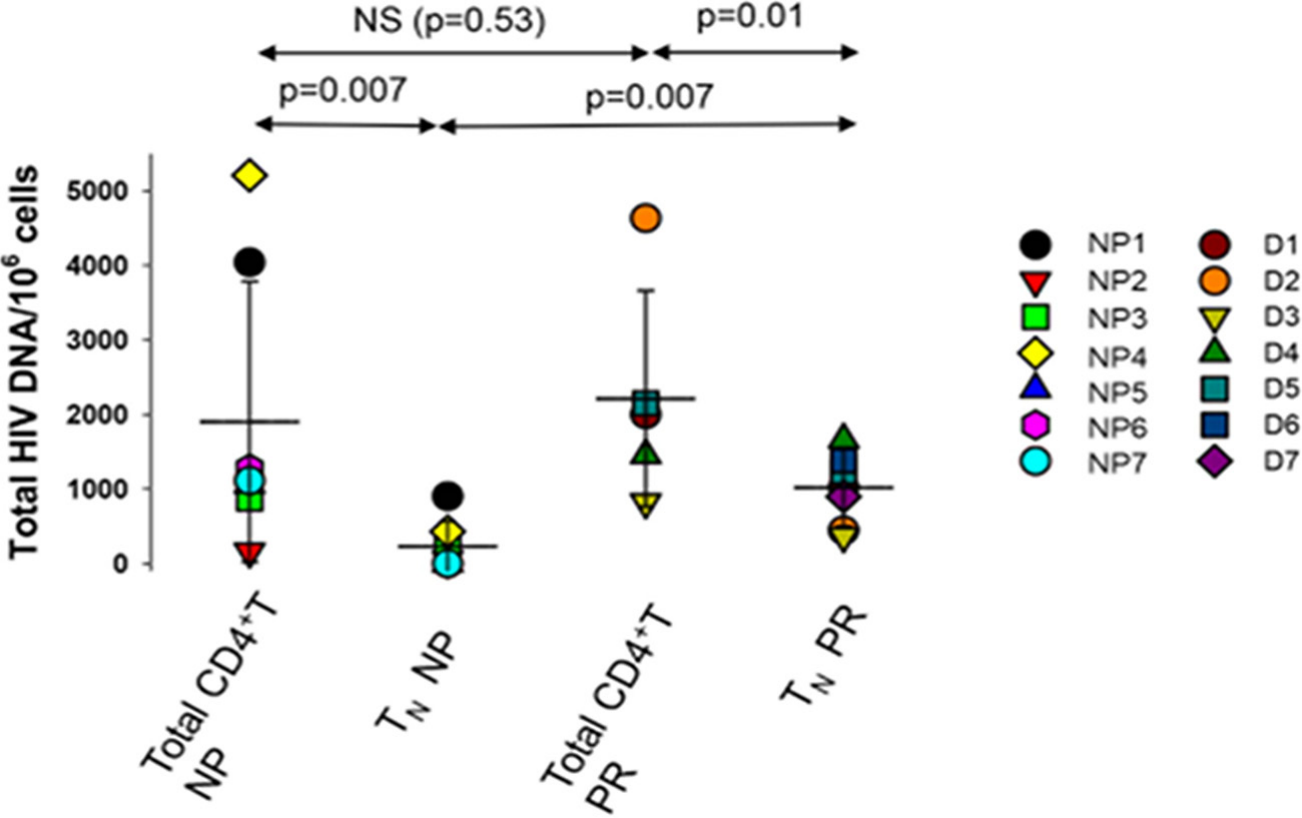
Quantification of total HIV-1 DNA in CD4^+^ total T cells and T_N_. DNA was quantified by qPCR as described in Materials and Methods. Each dot represents a unique donor. Statistical comparison was analyzed using a Wilcoxon matched-pairs signed-rank test. *P* < 0.05 was considered significant.

We have previously shown that APC from NP cannot *trans* infect autologous and heterologous CD4^+^ T cells and that this phenotype is under the control of cellular cholesterol homeostasis regulation (18, 19). Furthermore, this characteristic is present prior to infection with HIV-1, indicating that it is an innate, genetically controlled phenotype. If B cell-mediated *trans* infection of T_N_ is an important mechanism by which these cells become infected with HIV-1, then it is plausible that NP have a reduced or absent level of HIV-1 DNA in this CD4^+^ T cell subset. We therefore quantified the viral DNA reservoir in total CD4^+^ T cells and T_N_ from 7 NP not under antiretroviral therapy (ART) and 7 PR on ART (Fig. 7). The results show that we could not detect HIV-1 DNA in T_N_ from NP classified as elite controllers (EC), while a relatively low number of HIV-1 DNA copies were detected in long-term nonprogressors (LTNP) and viremic controllers (VC) (19). Overall, the average copy number of HIV-1 DNA in T_N_ from NP was lower than the number of copies detected in the 7 ART-suppressed PR (*P* = 0.007). NP and PR had similar levels of HIV-1 DNA copies when total CD4^+^ T cells were tested. Given that CD4^+^ T cells from NP as well as CD4^+^ T cells from PR are susceptible to direct, *cis* infection (19), the evidence supports the concept that the small amount of HIV-1 DNA detected is the result of direct infection. Taken together, these data support that individuals naturally able to control HIV-1 disease progression have a reduced or absent HIV-1 reservoir in their T_N_ population.

## DISCUSSION

Here, we show that B lymphocytes have the unique ability to *trans* infect CD4^+^ T_N_ *in vitro* with an R5-tropic HIV-1 laboratory strain (HIV-1^BaL^) and an R5 clinical isolate [HIV-1 BX08(92FR_BX08)], compared to myeloid DC. Prior studies have shown that T_N_ can be infected *in vitro* with CXCR4-tropic HIV-1 when pretreated with the chemokine CCL-19, the ligand for the CCR7 receptor, expression of which significantly increases during the acute phase of infection when the latent reservoir is established (9, 24). This treatment does not alter the activation or proliferation state of T_N_ and does not induce significant expression of the CCR5 coreceptor. Therefore, this model was used in our study to preserve the phenotype of the T_N_ population, which remained resistant to *cis* infection with an R5-tropic HIV-1 strain. Furthermore, exposure of T_N_ to B cells loaded with R5-tropic HIV-1^BaL^ during the coculture period did not induce significantly higher expression of the CCR5 receptor, thus excluding the possibility that the efficient *trans* infection we observed was the result of *in vitro* conditions. However, treatment of T_N_ with maraviroc under our culture conditions resulted in some inhibition of *trans* infection after 12 days of culture. This could be due to a waning effect of the drug *in vitro* or by *de novo* infection of cells expressing the CCR5 coreceptor later in the culture induced by the *in vitro* conditions, and being directly infected in *cis* by newly produced virus. Since this could also be occurring in the complex *in vivo* environment, it is possible that enhanced B cell-mediated *trans* infection of T_N_ is aided by expression of CCR5 on these cells once they start interacting with their cognate counterparts. Efficient transfer of HIV-1 to T_N_ mediated by B cells was also confirmed by detection of intracellular HIV-1 p24 by flow cytometry.

We have previously shown that activated B cells are able to bind and internalize HIV-1 into cytoplasmic vesicles through DC-SIGN and can *trans* infect CD4^+^ T cells for up to 2 days with high efficiency (15). Furthermore, we demonstrated that *trans* infection of total CD4^+^ T cells can be inhibited by treatment with anti-DC-SIGN MAb. Here, we confirmed that inhibition of DC-SIGN expression on B cells also impairs *trans* infection of T_N_. Notably, B cells do not support HIV-1 replication (15). Therefore, the second, *cis* infection phase in DC-mediated HIV-1 *trans* infection (26) is not applicable to B lymphocytes. Since cell-to-cell-mediated spread of HIV-1 is several orders of magnitude more efficient than direct *cis* infection of target cells (17), this mode of virus dissemination could have a significant role in HIV-1 pathogenesis, particularly in T cell-APC-dense anatomical compartments (16, 27). We propose that this *trans* infection process is likely intertwined with basic immunologic interactions of B lymphocytes and T_N_. Indeed, B cells were recently described as having a broad role in the development of T_N_ (28). The interaction between B cells and CD4^+^ T cells thus goes beyond the classical initiation of antigen-specific B cell differentiation into antibody-producing plasma cells. In fact, evidence suggests that B cells are necessary and sufficient to prime and activate T_N_ in response to virus-like particles (28). Thus, unique features of interactions between B cells and T_N_ could drive the transfer and replication of HIV-1. In T-dependent B cell immune responses, antigen-engaged B cells must find their cognate helper T cells to initiate the progression of B cell immune responses. However, within the lymph node follicle, B cells move continuously to survey the subcapsular (SCS) macrophages for surface-displayed antigens (29, 30) and are also receiving survival signals from fibroblastic reticular cells (FRC) (31) such as the B cell activator BAFF, which has been shown to activate B cells to express DC-SIGN (32). These B cells are positioned to capture HIV-1 either as free virus entering through the afferent lymph vessel or through sampling of SCS macrophages (33), which have poor endocytic capacity and limited degradative ability (30). This ultimately prevents them from efficiently degrading HIV-1. In this scenario, subcapsular B cells activated through a T-independent mechanism are perfectly positioned to capture HIV-1 particles while surveying the environment for their specific antigen.

Upon encounter with antigen, signaling via the B cell receptor (BCR) starts the sequence of events that will bring the antigen-specific B cells to the follicle-T zone boundary where they search for their cognate CD4^+^ T cell among the CD4^+^ T helper cells or T_N_ residing there (34). This interaction provides an opportunity for transfer of HIV-1 that has been captured by B cells to the CD4^+^ T cells. This interaction at the follicle-T cell zone interface of lymph nodes can last from several minutes to an hour (35, 36) and requires the interaction of integrins, such as LFA1 on T helper cells interacting with ICAM-1 or ICAM-2 on B cells, as well as costimulatory molecule CD86 signaling of CD28. Crucially, these interactions are stabilized by the antigenic peptide presented by B cell-expressed major histocompatibility complex (MHC) class II. Notably, it is known that B cells are superior to DC in capturing high doses of cognate antigen through high-affinity antigen-specific receptors, therefore rendering B cell-mediated antigen stimulation more efficient than that by DC (33). B cells capture antigen with high affinity through the BCR, allowing for even a low concentration of antigen to result in high internalization and subsequent presentation to T cells (37) and upregulation of the costimulatory molecule CD86 expression. On the other hand, DC capture antigen through nonspecific binding, requiring higher levels of antigen to induce a CD4^+^ T cell response. Moreover, the interaction between DC and T_N_ is not prolonged, resulting in a lower chance of virus being transferred, even though activated B cells and DC express costimulatory molecules involved in the formation of the immunological synapse. Thus, the unique features of the interactions between B and T_N_ could drive the transfer of HIV-1 to T_N_ with higher efficiency than that of DC. It has been reported that high expression of Siglec-1 (CD169) on DC matured with lipopolysaccharide (LPS) and type I interferon (IFN) significantly contributes to *trans* infection (38, 39), and its importance in retrovirus infections has been shown in a mouse model (40). The different conditions under which DC can be matured and the effect on their ability to *trans* infect human T_N_ are beyond the scope of our study, but it is possible that DC matured under different stimuli could be able to infect T_N_ through *trans* mechanisms.

Although T_N_ represent the more abundant fraction of CD4^+^ T cells, most studies of the latent HIV-1 reservoir have focused on memory T cells because they harbor the highest levels of HIV-1 DNA in people under ART. We and others (9, 12, 22) have shown that although the frequency of HIV-1 infection in these cells is lower than other subsets, as much or more virus is produced by these cells after treatment with LRA. This is true also when T_N_ isolated from HIV-1-infected individuals under ART are exposed to LRA. Paradoxically, although T_N_ do not express the HIV-1 coreceptor CCR5, they harbor CCR5-tropic virus *in vivo*. Therefore, an understanding on how this subset of CD4^+^ T cells becomes infected could provide important clues in the development of strategies to thwart the early establishment of HIV-1 infection.

As we have previously shown, efficient APC-mediated *trans* infection is regulated by APC membrane cholesterol content and is related to the control of HIV-1 disease progression (18, 19). In fact, APC derived from HIV-1-infected NP have an innate inability to *trans* infect CD4^+^ T cells, and this phenotype can be reversed by replenishing cell membrane cholesterol. On the other hand, APC from HIV-1-infected individuals with progressing disease, i.e., PR, mediate efficient HIV-1 *trans* infection (18, 19). Here, we quantified the viral reservoirs in total CD4^+^ T cells and T_N_ from NP and PR in the Pittsburgh clinical site of the MACS/WIHS Combined Cohort Study (MWCCS). Notably, while the PR studied here were under suppressive ART, all the NP tested were therapy naive at the time of the study. We could not detect viral DNA in NP classified as EC, while a significantly smaller number of HIV-1 DNA copies was detected in LTNP and VC than in PR. These data strongly suggest that the altered ability to *trans* infect CD4^+^ T cells in NP results in a small or negligible pool of latently infected T_N_, with HIV-1 DNA levels even lower than those detectable in patients under suppressive ART, thus contributing to the maintenance of the NP phenotype. Although limited in scope, our findings are also consistent with that observed in the French Virological and Immunological Studies in Controllers After Treatment Interruption (VISCONTI) cohort of individuals who received ART within 10 weeks of primary infection (41), where viremia was controlled for 24 months post-treatment interruption. In that cohort, HIV-1 DNA was detected in T_N_ of only 2 out of 11 patients, while the other T cell subsets harbored comparable levels of HIV-1 DNA. Our present study suggests that early, B cell-mediated *trans* infection could be an important mechanism by which HIV-1, regardless of its basic cell tropism, establishes infection in T_N_. We propose an additional role for B cell-mediated *trans* infection, not only as an efficient means to spread HIV-1 to CD4^+^ T cells but as the driver in establishing the HIV-1 reservoir in T_N_ and potential consequent control of HIV-1 disease progression.

## MATERIALS AND METHODS

### Ethics statement.

Biological samples were acquired and studied from consenting individuals according to University of Pittsburgh International Review Board-approved protocols. All recruited participants were over the age of 18 and provided written consent prior to sample collection or use.

### Cohort.

Experiments were performed using peripheral blood mononuclear cells (PBMC) obtained from anonymous donors (HIV-1 negative, *n* = 6) from the Pittsburgh blood bank (Vitalant Pittsburgh) or archived PBMC obtained from 7 HIV-1-infected NP and 7 HIV-1-infected PR enrolled in the Pittsburgh portion of the MACS/WIHS Combined Cohort Study (MWCCS). The NP cohort consisted of 3 NP (LTNP, CD4^+^ T cell counts >500 cells/mm^3^ over >7 years postinfection), 3 elite controllers (EC, undetectable viral load >7 years postinfection), and 1 viremic controller (VC, at least two viral load measures below 2,000 copies of HIV-1 RNA/ml).

### Generation of CD4^+^ T cell subsets.

Naive and central memory CD4^+^ T lymphocytes were obtained from resting PBMC by magnetic bead negative selection according to the manufacturer’s instructions (Miltenyi Biotech). CD4^+^ T_N_ were defined as CD45RA^+^ CCR7^+^ CCR5^−^, while CD4^+^ T_CM_ were defined as CD45RA^−^ CCR7^+^ CCR5^+^. The relative purity of the separated fractions was determined by flow cytometry.

### Cell isolation and culture.

CD4^+^ T lymphocytes, B lymphocytes, and CD14^+^ monocytes were positively selected from PBMC using anti-CD4, -CD19, or -CD14 monoclonal antibody (MAb)-coated magnetic beads (Miltenyi Biotech). Immature DC (iDC) were derived from CD14^+^ monocytes cultured with 1,000 U/ml granulocyte-macrophage colony-stimulating factor (GM-CSF; Miltenyi Biotech) and 1,000 U/ml recombinant human interleukin-4 (rhIL-4) for 5 days in AIM-V medium, with additional GM-CSF and rhIL-4 added on day 3. Mature DC (mDC) were derived from iDC by the addition of 0.1 μg/ml trimeric CD40L (Enzo) on day 5 and cultured for an additional 2 days. Prior to coculture, CD4^+^ T cells and B cells were activated for 48 h with 10 U/ml IL-2 (Roche) and 2 μg/ml phytohemagglutinin (PHA; Sigma) or 1,000 U/ml rhIL-4 and 0.1 μg/ml trimeric CD40L (Enzo), respectively. CD4^+^ T_N_ or T_CM_ were treated with either 100 nM CCL-19 (R&D Systems) or 10 U/ml IL-2 and 2 μg/ml PHA for 48 h as previously described (12, 15).

### Cell phenotyping.

Cells were assessed for surface protein expression by flow cytometry. B cell + T_N_ and DC + T_N_ cocultures or T_N_ alone were incubated with LIVE/DEAD fixable Aqua viability cell stain kit (Invitrogen) for 20 min and then incubated with MAb against CD3 (allophycocyanin-H7), CD4 (V450), CCR5 (phycoerythrin [PE]), CD45RA (PE-CF594), CCR7 (allophycocyanin), and CD27 (fluorescein isothiocyanate [FITC]) for 20 min. Cells were fixed with 1% paraformaldehyde (PFA), acquired with a BD LSR Fortessa and analyzed with FlowJo V10. The gating strategy is described in Fig. S1 in the supplemental material.

Virus stock titration and experimental p24 measurements were acquired by enzyme-linked immunosorbent assay (ELISA) using the HIV-1 p24 antigen capture immunoassay (Leidos Biomedical Research, Frederick National Laboratory for Cancer Research) per the manufacturer's instructions. HIV-1 Gag p24 was also evaluated in *trans* and *cis* infection cocultures by flow cytometry. Briefly, cocultures were harvested and incubated with LIVE/DEAD fixable Aqua viability stain kit (Invitrogen) for 20 min and then incubated for surface staining with MAb against CD3 (allophycocyanin-H7), CD4 (PE), and CD19 (PE-CF594) for 20 min. Cells were then permeabilized with PermII buffer (BD) for 20 min, washed, incubated with anti-HIV-1 p24 antibody Kc57-FITC (Coulter) for 20 min, washed, and resuspended in 1% PFA prior to analysis with a BD LSR Fortessa. Acquired data were analyzed with FlowJo V10. The gating strategy is described in Fig. S2.

10.1128/mBio.02998-20.2 FIG S2 Gating strategy to determine intracellular HIV-1 Gag p24. Lymphocytes were gated first based on forward and side scatter, followed by doublet event exclusion, then by exclusion of dead cells (Aqua dye positive). CD4^+^/HIV-1 Gag p24^+^ cells and CD4^neg^/HIV-1 Gag p24^+^ cells were then gated within the CD4^+^/CD3^+^ population. Download FIG S2, TIF file, 1.3 MB. Copyright © 2021 Gerberick et al. 2021 Gerberick et al. https://creativecommons.org/licenses/by/4.0/ This content is distributed under the terms of the Creative Commons Attribution 4.0 International license.

### *Trans* and *cis* infection.

R5-tropic HIV-1^BaL^, grown in and purified from PM1 cells (42) (American Type Culture Collection), was used for *cis* and *trans* infection experiments. A patient isolate, R5-tropic HIV-1 BX08(92FR_BX08), used in *trans* and *cis* experiments was obtained from the NIH AIDS Reagents Program, Division of AIDS, NIAID, NIH [HIV-1 BX08(92FR_BX08) virus (catalog no. 11420) from Victoria Polonis] (23).

**(i) *Trans* infection.** 1 × 10^6^ APC were incubated with a low concentration of HIV-1^BaL^ or HIV-1BX08 (10^−3^ MOI) for 2 h at 37°C and then washed 3 times with cold medium. Virus-loaded APC were cocultured with autologous CD4^+^ T cell targets at a 1:10 effector/target ratio in R10 medium.

**(ii) *Cis* infection.** Activated CD4^+^ T cells or T_N_ were incubated with a high concentration of HIV-1^BaL^ or HIV-1BX08 (10^−1^ MOI) for 2 h at 37°C, washed 3 times with cold medium, and cultured independently. For both *trans* and *cis* infection, HIV-1 Gag p24 levels were quantified in cell-free supernatants at days 4, 8, and 12 post coculture. In some experiments, stimulated B cells were incubated with 20 μg/ml anti-DC-SIGN MAb (clone 120507; R&D Systems) or mouse IgG (R&D Systems) for 30 min at 4°C prior to incubation with virus. In some experiments, T_N_ were incubated with maraviroc (1 μM) as previously described (25).

### Reactivation of latent HIV-1 from APC-T_N_ cocultures.

Eight days after the start of APC-T_N_ cocultures, cells were treated with 10 nM phorbol myristate acetate (PMA; Sigma-Aldrich) and 10 μg/ml PHA (PMA-PHA). Supernatants were collected at days 11, 14, and 17. Levels of HIV-1 Gag p24 were then tested by ELISA. Parallel untreated cultures were used as control.

### Quantification of total HIV-1 DNA.

Total HIV-1 DNA in CD4^+^ T cells and T_N_ was quantified as described previously (43).

### Statistics.

Data were tested by the Shapiro-Wilk normality test and analyzed by one-way analysis of variance (one-way ANOVA) followed by Student *t* tests. GraphPad Prism 7.0 software was used for statistical analysis.
