# Supplementary figures and images for: B Lymphocytes, but Not Dendritic Cells, Efficiently HIV-1 Trans Infect Naive CD4+ T Cells: Implications for the Viral Reservoir
Source: mBio. 2021 Mar 9;12(2):e02998-20. doi: 10.1128/mBio.02998-20 (PMC8092276; doi:10.1128/mBio.02998-20)

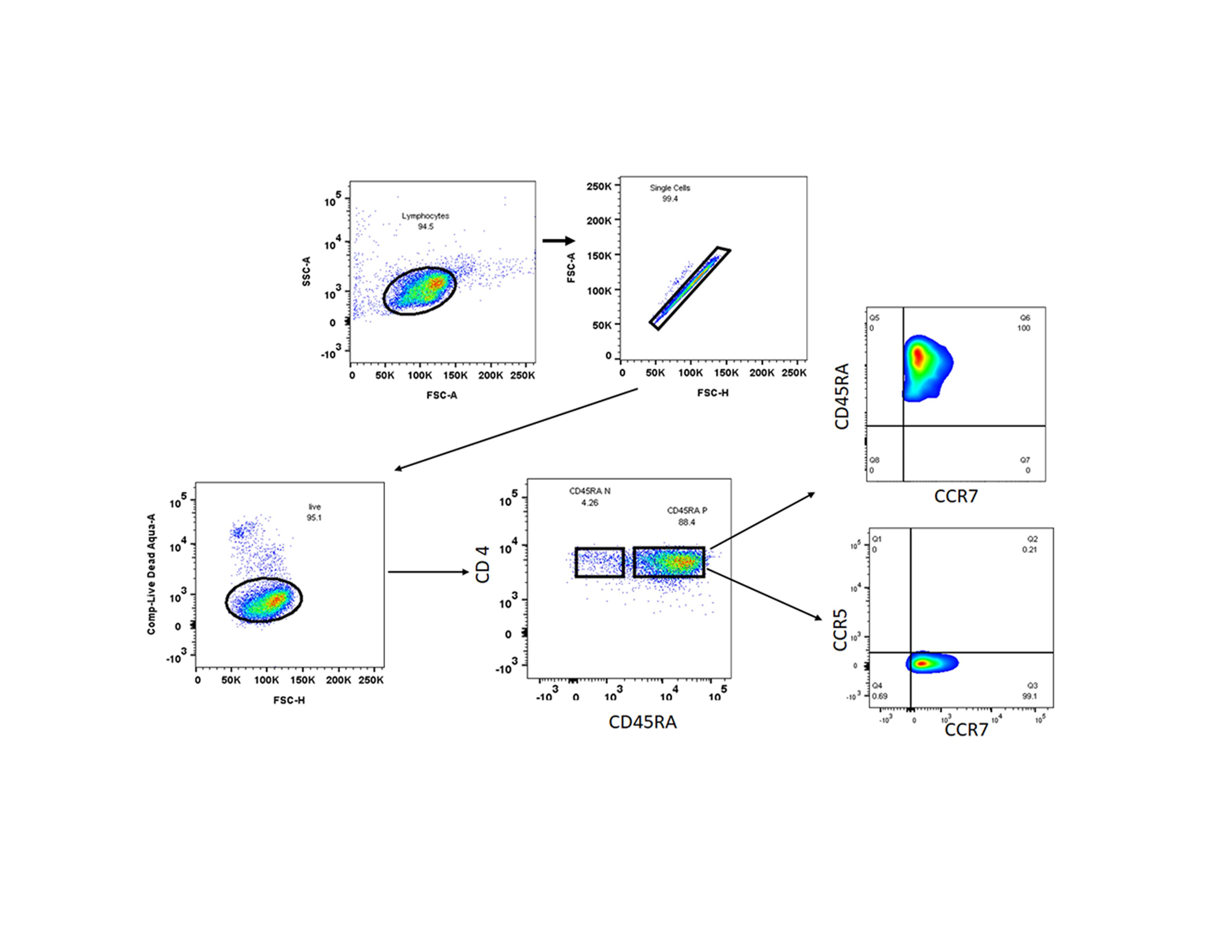

Supplement: FIG S1 [file mBio.02998-20-sf001.tif]

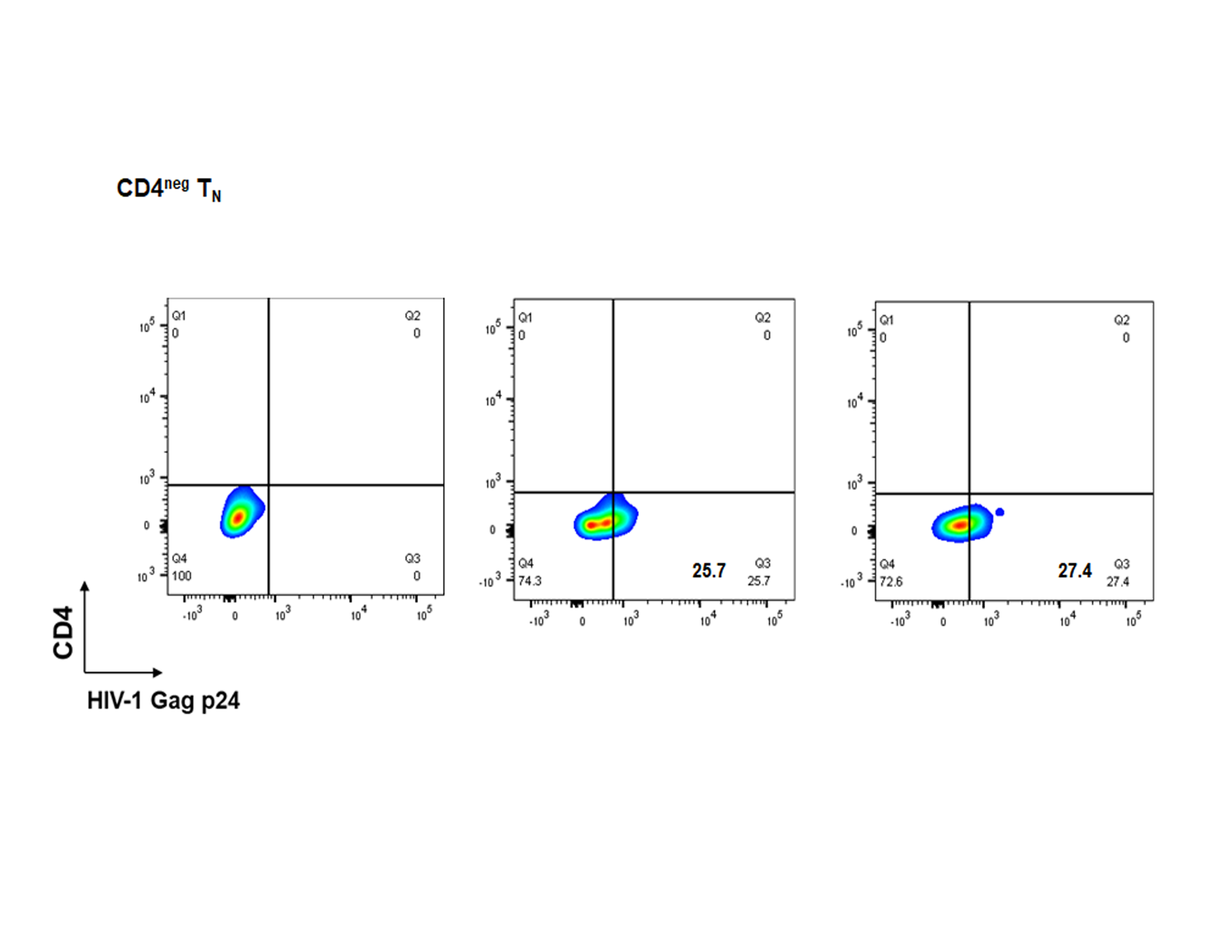

Supplement: FIG S3 [file mBio.02998-20-sf003.tif]

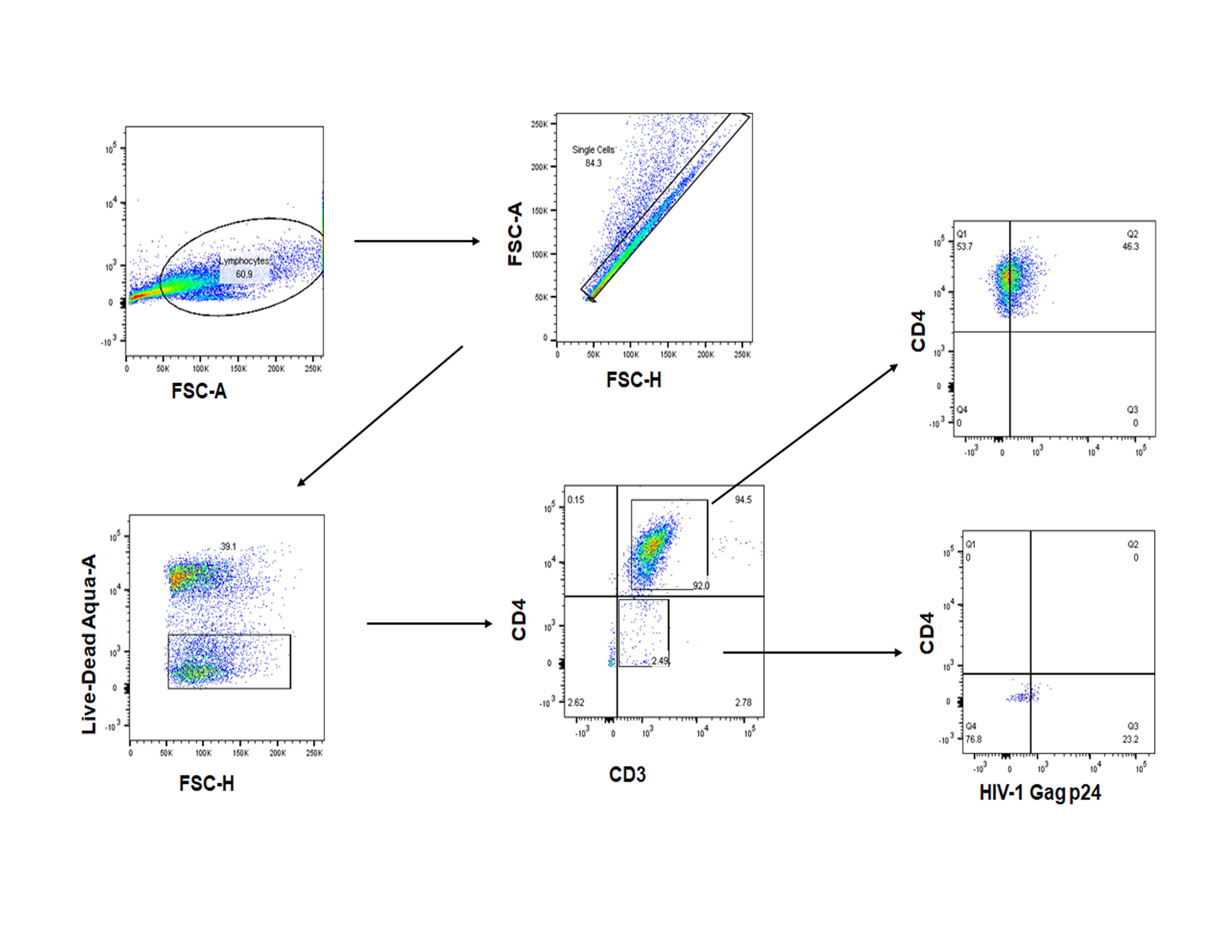

Supplement: FIG S2 [file mBio.02998-20-sf002.tif]
